# Postmortem gene expression profiles in the habenulae of suicides: implication of endothelial dysfunction in the neurovascular system

**DOI:** 10.1186/s13041-022-00934-7

**Published:** 2022-05-25

**Authors:** Hyun Jung Kim, Hyeijung Yoo, Ji Yeon Kim, Soo Hyun Yang, Hyun Woo Lee, Heon-Jeong Lee, Gi Hoon Son, Hyun Kim

**Affiliations:** 1 Department of Biomedical Sciences, College of Medicine, Korea University, Seoul, 02841 Republic of Korea; 2 Department of Anatomy and Neuroscience, College of Medicine, Korea University, Seoul, 02841 Republic of Korea; 3 Department of Legal Medicine, College of Medicine, Korea University, Seoul, Republic of Korea; 4 Department of Psychiatry, Korea University College of Medicine and Anam Hospital, Seoul, Republic of Korea

**Keywords:** Depression, Suicide, Habenula, Postmortem, Gene expression, Transcriptome

## Abstract

The habenula (Hb) is an epithalamic structure that links multiple forebrain areas with the mid/hindbrain monoaminergic systems. As an anti-reward center, it has been implicated in the etiology of various neuropsychiatric disorders, particularly those associated with dysregulated reward circuitry. In this regard, Hb has been proposed as a therapeutic target for treatment-resistant depression associated with a higher risk of suicide. Therefore, we aimed to gain insight into the molecular signatures of the Hb in association with suicide in individuals with major depression. Postmortem gene expression analysis identified 251 differentially expressed genes (DEGs) in the Hb tissue of suicides in comparison with Hb tissues from neurotypical individuals. Subsequent bioinformatic analyses using single-cell transcriptome data from the mouse Hb showed that the levels of a subset of endothelial cell-enriched genes encoding cell–cell junctional complex and plasma membrane-associated proteins, as well as the levels of their putative upstream transcriptional regulators, were significantly affected in suicides. Although our findings are based on a limited number of samples, the present study suggests a potential association of endothelial dysfunction in the Hb with depression and suicidal behavior.

**Supplementary Information:**

The online version contains supplementary material available at 10.1186/s13041-022-00934-7.

The habenula (Hb) is an epithalamic structure located at the dorsomedial posterior end of the thalamus in mammals. It serves as a neuroanatomical bridge, linking multiple forebrain structures such as the frontal cortex, basal forebrain, and hypothalamus, with monoaminergic nuclei in the mid- and hindbrain. The Hb has been recently recognized for its role in encoding aversive stimuli as an anti-reward center, and its implications in neuropsychiatric disorders associated with dysregulated reward circuitry, such as depressive disorders, schizophrenia, and substance use disorders [1, 2]. Notably, Hb has been proposed as a therapeutic target for treatment-resistant major depressive disorder with a higher risk of suicide [3, 4], and its altered connectivity has been associated with suicidal ideation [5]. Our previous study also revealed that several genes involved in cholinergic signaling exhibit decreased mRNA expression levels in Hb tissue of suicides [6]. To gain systemic insights into the molecular signatures of human Hb underlying suicide with major depression, the present study examined genome-wide gene expression profiles in the Hb from suicide completers compared to unaffected controls.

For this purpose, we compared postmortem gene expression patterns between groups using a microarray platform. All subjects were male Caucasians, and groups (suicides and controls, n = 10 for each group) were matched for age, postmortem interval (PMI), and pH; Age: 44.20 ± 3.35 years (mean ± SE) for controls and 40.50 ± 4.35 for suicides; PMI: 26.45 ± 4.95 h for controls and 36.10 ± 4.32 for suicides; pH: 6.671 ± 0.047 for controls and 6.427 ± 0.095 for suicides (see Additional file 1 for experimental procedures and Additional file 2: Table S1 for more detailed subject information). Among 20,085 detected genes, RNA expression levels of 796 were significantly different between groups (p < 0.05), and 251 genes at the criteria of *p* < 0.05, and > 1.2-fold change (FC) were selected as differentially expressed genes (DEGs) for subsequent bioinformatic analyses (Additional file 2: Table S2). *ARAP3*, *PHLDB2*, *PAQR5*, *PMAIP1*, and *GPR116* were the top-five upregulated DEGs, and *NPNT*, *HSPA1A/B*, *SLC22A4*, *PAX3*, and *UBC* were the most downregulated genes (Fig. 1a). We then compared our DEGs with disease-associated genes curated in the PsyGeNET database [7] and found that 28 DEGs are implicated in several psychiatric disorders (Fig. 1b). Gene-disease associations clearly show that these genes are associated with depressive disorder, bipolar disorder, substance abuse (alcohol, cannabis, or cocaine), and schizophrenia (Fig. 1c).

**Fig. 1:**
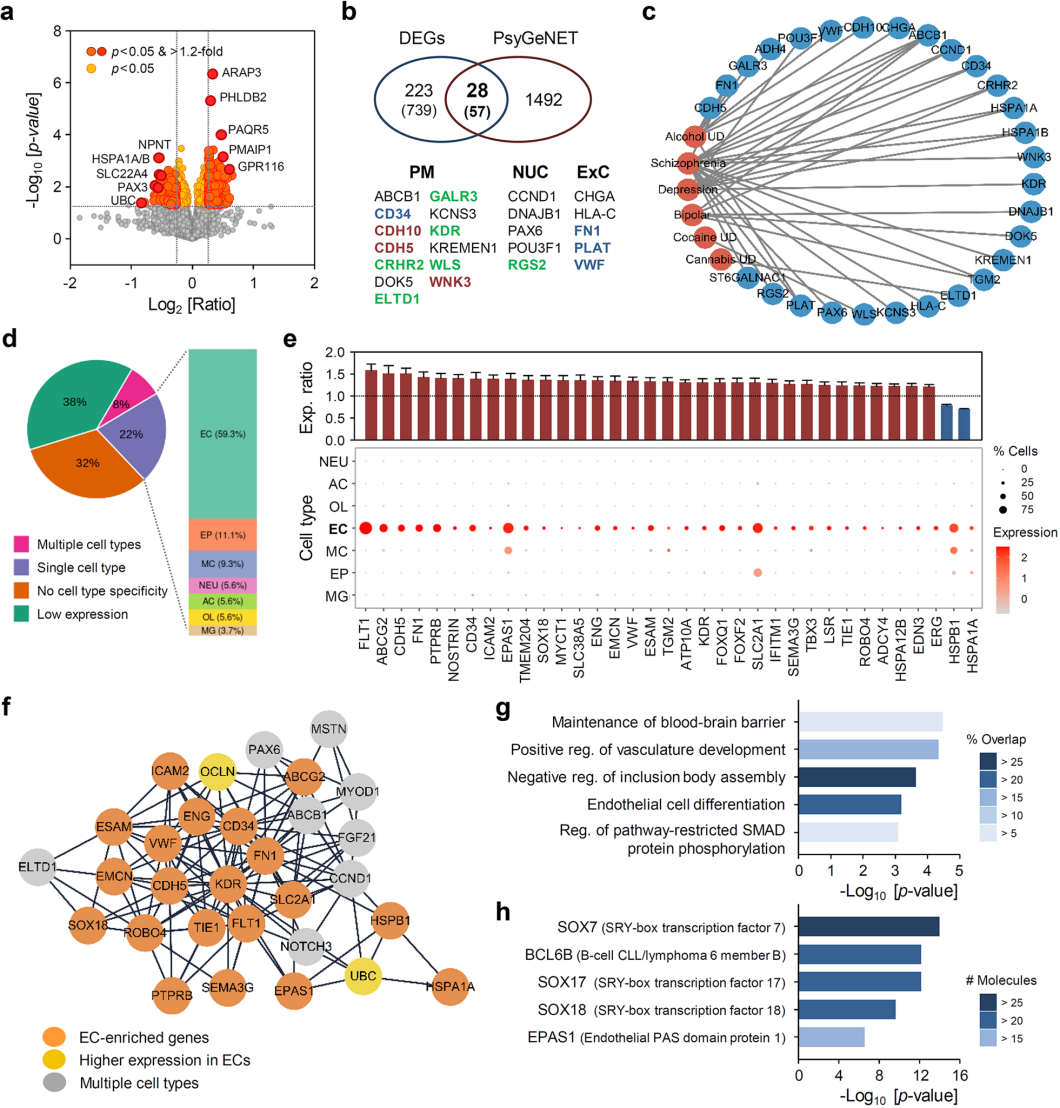
Characterization of differential gene expression profiles in the suicidal habenula (Hb) with major depression. **a** Volcano plot for Hb gene expression levels in suicides and unaffected controls. Differentially expressed genes (DEGs), defined by p < 0.05, are shown in yellow (FC < = 1.2) or orange/red (FC > 1.2) circles. **b** Venn diagram for comparison of the 251 main DEGs and psychiatric disease-associated genes curated by the PsyGeNET database. Numerals in parenthesis indicate numbers of genes with p < 0.05 between groups with regardless of FC. Disease-associated genes are listed below the Venn diagram and categorized into several groups according to their major subcellular localization (PM: plasma membrane; NUC: nucleus; ExC: extracellular space). Genes in red characters constitute cell–cell junctions; genes in blue represent components of or interacting with extracellular matrix; and genes in green are membrane receptors and their accessory proteins. **c** Association between the PsyGeNET disease terms (red symbols) and disease-associated DEGs (blue). **d** Summary of cell-type enrichment analysis of the main 251 DEGs (EC: endothelial cell; EP: ependymal cell; MC: mural cell; NEU: neuron, AC: astrocyte, OL: oligodendrocyte lineage cell, MG: microglia). **e** mRNA expression levels of EC-enriched DEGs in suicides relative to mean expression levels of controls as expressed in mean ± SE (upper) and dot plot showing gene expression across cell types (lower). **f** Subcluster of the top-30 genes selected using maximum neighborhood component method from protein–protein interaction (PPI) networks constructed using the 251 DEGs with confidence cut-off of 0.4. **g** Featured EC-related processes among top-10 gene ontology biological process (GO-BP) terms enriched in DEGs are expressed by p-value and % overlap with the entire gene list of each GO-BP term. Percent overlaps are color-coded as indicated. **h** Top-5 co-expressed transcription factors (TFs) with DEGs suggested by the ARCHS4 TF co-expression database are expressed by p-value and number of co-expressed genes with each TF. Numbers of co-expressed DEGs are grouped and color-coded as indicated

Next, we categorized the DEGs by cell type enrichment in their expression using single-cell level gene expression profiles obtained from murine Hb [8]. Interestingly, cell type enrichment analysis raised a possibility that endothelial cells (ECs) constituting brain microvasculature could be one of the most affected cell types in the suicidal Hb; 59.3% of DEGs with cell-type-restricted expression were EC-enriched genes in the mouse Hb region (Fig. 1d and Additional file 2: Table S3). Among 155 DEGs found in the murine Hb dataset, 47 DEGs were highly enriched or even exclusively expressed in ECs, and most of them, except *HSPA1A* and *HSPB1*, exhibited significantly increased expression levels in the suicidal Hb (Fig. 1e, upper panel). The EC-enriched DEGs included genes encoding key components of cell–cell junctions such as *CDH5*, *ESAM*, *LSR*, and *OCLN*, along with those encoding plasma membrane-associated proteins such as *ABCG2*, *ADCY4*, *ATP10A*, *CD34*, *FLT1*, *ICAM2*, *KDR*, *PTPRB*, *SEMA3G*, *SLC2A1*, *SLC38A5*, and *TIE1*, implying possible alterations in endothelial functions, particularly involving blood–brain barrier (BBB) permeability [9]. Protein–protein interaction (PPI) analysis also supported this notion. A PPI network was constructed to elucidate functional interactions between DEGs (221 nodes and 272 edges), and subsequent subcluster network analysis proposed the top-30 genes consisting of 20 EC-enriched genes and 2 genes with relatively higher expression levels in ECs, which also included genes linked with BBB permeability (Fig. 1f).

In good agreement with cell-type enrichment and PPI analyses for the identified DEGs, gene ontology (GO) analysis also suggested endothelial dysfunction. Among the featured GO biological processes, three terms such as “maintenance of BBB,” “positive regulation of vasculature development” and “endothelial cell differentiation,” are intricately linked with the roles of ECs in controlling BBB and/or microvasculature in brain tissues (Fig. 1G and Additional file 2: Table S4). Concerted regulation of EC-enriched genes suggested that a subset of upstream transcription factors (TFs) required for EC differentiation and function may underlie suicide-associated alterations in Hb gene expression profiles. To test this idea, we conducted gene-TF co-expression analysis using the ARCHS4 database (https://maayanlab.cloud/archs4/) and found that putative target genes of several EC-specific TFs were significantly enriched among the DEGs (Fig. 1h and Additional file 2: Table S5). Among the top-five co-expressed TFs, *BCL3B*, *SOX18* and *EPAS1* mRNA expression levels were significantly higher in the suicides than controls (Additional file 2: Table S2). It should be noted that the SOXF subgroup of the SOX (SRY-box transcription factor) family such as SOX7, SOX17, and SOX18 is well known as EC-enriched TFs required for vasculogenesis [10] and plays a major role in controlling BBB permeability through reciprocal crosstalk with canonical Wnt/β-catenin signaling [11]. In addition, another EC-enriched TF, EPAS1 (endothelial PAS domain protein 1) was predicted to be activated in the suicidal Hb (Fig. 1e); its mRNA expression is known to be inducible in response to hypoxic/ischemic stimuli in brain tissues [12]. One may suppose that the altered expression of EC-specific genes may represent different EC proportions between groups. However, it should be noted that the mRNA expression levels of well-established EC markers such as *CLDN5*, *ICAM1*, *VCAM1*, *PTPRC*, *FLT4,* and *TEK* were not significantly different between the groups (Additional file 2: Table S6). Therefore, it is more reasonable that the differential mRNA expression of a subset of EC-enriched genes may represent altered microvasculature functions in the Hb.

Several postmortem studies have implicated brain endothelial dysfunction in discrete brain areas including the prefrontal cortex, nucleus accumbens, and amygdala in the etiology of certain types of depressive disorders [13, 14]; altered gene expression profiles responsible for impaired BBB resistance and enhanced angiogenic signaling have been particularly linked to depressive disorders [13]. As the Hb is one of the brain regions where ECs are abundant [15], it could represent a major site of action of EC-enriched and psychiatric disease-associated genes such as *CD34*, *CDH5*, *FN1*, *HSPA1A*, *KDR*, *TGM2*, and *VWF* (Fig. 1b and 1e). Therefore, it is plausible that DEGs promoting BBB permeability and vasculature development in the Hb may underlie depressive phenotypes, including suicidal behavior.

In conclusion, the present study identified a set of genes whose mRNA expression is significantly altered in the Hb of suicides, and suggests that endothelial dysfunction, presumably linked to Hb microvasculatures and BBB integrity, may play a role in the etiology of suicide associated with major depression. Although the present study is primarily based on transcriptome profiles of bulk Hb tissues with a limited number of samples, we provide putative molecular and cellular targets, implying possible importance of Hb ECs in mood regulation and suicidal behavior. The functional consequences of EC dysfunction on neuroglial properties in the Hb, as well as emotional abnormalities, should be further delineated.

## Supplementary Information

**Additional file 1.** Materials and methods including subject information, RNA isolation, microarray analysis, and subsequent bioinformatic analyses.

**Additional file 2: Table S1.** Subject information; **Table S2.** Full list of the main 251 DEGs; **Table S3.** Cell-type enrichment analysis using single-cell level gene expression profiles obtained from murine habenula; **Table S4.** Top-10 GO-BP terms and related genes; **Table S5.** Top-10 ARCHS4 co-expressed TFs and putative target DEGs; **Table S6.** List of EC markers that did not exhibit significant changes in mRNA expression levels.

## Data Availability

The datasets used and analyzed in the present study are available from the corresponding authors upon reasonable request. Raw microarray data have been submitted to the Gene Expression Omnibus (GEO) repository (Accession number: GSE199536).

## Abbreviations

Hb: Habenula
DEG: Differentially expressed gene
FC: Fold change
EC: Endothelial cell
EP: Ependymal cell
MC: Mural cell
NEU: Neuron
AC: Astrocyte
OL: Oligodendrocyte lineage cell
MG: Microglia
BBB: Blood–brain barrier
PPI: Protein–protein interaction
GO: Gene Ontology
GO-BP: GO biological process
TF: Transcription factor
