# Supplementary material for: Postmortem gene expression profiles in the habenulae of suicides: implication of endothelial dysfunction in the neurovascular system
Source: Mol Brain. 2022 May 25;15:48. doi: 10.1186/s13041-022-00934-7 (PMC9134578; doi:10.1186/s13041-022-00934-7)
Supplement: Supplementary file 1 — Additional file 1. Materials and methods including subject information, RNA isolation, microarray analysis, and subsequent bioinformatic analyses. [file 13041_2022_934_MOESM1_ESM.docx]

**Postmortem gene expression profiles in the habenulae of suicides: Implication of endothelial dysfunction in the neurovascular system**

Hyun Jung Kim^1,2,5^, Hyeijung Yoo^1,2,5^, Ji Yeon Kim^1,3^, Soo Hyun Yang^1,2^, Hyun Woo Lee^1,2^, Heon-Jeong Lee^4^, Gi Hoon Son^1,3*^ & Hyun Kim^1,2*^

^1^Department of Biomedical Sciences, ^2^Department of Anatomy and Neuroscience, and ^3^Department of Legal Medicine, College of Medicine, Korea University, Seoul, Republic of Korea

^4^Department of Psychiatry, Korea University College of Medicine and Anam Hospital, Seoul, Republic of Korea
^5^These authors contributed equally.

*Correspondence: Hyun Kim, M.D., Ph.D. and Gi Hoon Son, Ph.D.

Hyun Kim, M.D., Ph.D.

Department of Anatomy and Neuroscience, College of Medicine, Korea University, Seoul 02841, Republic of Korea (E-mail: [kimhyun@korea.ac.kr](mailto:kimhyun@korea.ac.kr))

Gi Hoon Son, Ph.D.

Department of Biomedical Sciences, College of Medicine, Korea University, Seoul 02841, Republic of Korea (E-mail: [songh@korea.ac.kr](mailto:songh@korea.ac.kr))

**Additional File 1: Materials and Methods**

**Subjects.** Brain tissues were prepared in the Douglas Bell Canada Brain Bank (Douglas Mental Health University Institute, Verdun, Quebec, Canada; <https://douglasbrainbank.ca/>). To avoid prolonged agonal states, both cases and controls recruited to the bank did not undergo resuscitation or medical intervention. Brains were collected after consent was obtained from the next of kin. To obtain diagnostic information, families were re-contacted a few months following recruitment to undergo a series of structured interviews with the person best acquainted with the deceased, a process commonly known as psychological autopsy. Interviews were supplemented with medical charts or other records, such as files from the police, coroner, or social services. Following the interviews, clinical vignettes were produced and assayed by a panel of clinicians to generate the Diagnostic and Statistical Manual of Mental Disorders (DSM) IV diagnostic criteria. In addition, samples from brain tissue, peripheral blood, and urine were used for toxicological analysis. Once enrolled, brains were sectioned, rapidly frozen in isopentane, and then stored at −80 °C.

**Preparation of habenula tissues** For this study, Hb tissues were prepared from 10 suicide subjects diagnosed with major depression and 10 psychiatrically healthy control subjects. All subjects were male Caucasians, and groups (suicides and controls) were matched for age, pH, and postmortem interval (PMI). Hb was carefully dissected, as described in our previous study [1]. Briefly, the human brain was divided into left and right hemispheres, and the meninges of the hemispheres were carefully removed. The trunk from the hemisphere was separated with a scalpel between the mammillary body and the superior colliculus. The pH of the sample was measured using the cerebellum. To make slabs, the brain hemispheres were placed with the median face down on a cutting plate and cut coronally into 18–20 pieces. Since Hb typically exists in the 11th or 12th slabs, Hb was gently removed from the frozen slab using a burin with a round-shaped tip.

**Microarray and gene enrichment analyses** RNAs longer than approximately 200 nucleotides were purified from each Hb tissue using RNeasy Mini kits according to the manufacturer’s instructions (Qiagen GmbH, Hilden, Germany). RNA integrity was evaluated using an Agilent 2100 Bioanalyzer (Agilent, Santa Clara, CA, USA). For each sample, 200 ng of total RNA was amplified, labeled, and analyzed with GeneChip Human Gene 2.0 ST arrays (Affymetrix, Santa Clara, CA, USA) according to the manufacturer’s protocol. Genes exhibiting > 1.2-fold changes at p < 0.05, as determined by Student’s *t*-test, were regarded as differentially expressed genes (DEGs). The datasets used and analyzed in the present study are available from the corresponding authors upon reasonable request. Raw microarray data have been submitted to the Gene Expression Omnibus (GEO) repository (Accession number: GSE199536).

**Identification of psychiatric disease-associated genes** Associations between DEGs and psychiatric diseases were identified using the PsyGeNET database [2]. Enrichment for each psychiatric disease was analyzed with the PsyGeNET2r package. The evidence index of PsyGeNET is expressed as the number of pieces of evidence supporting the existence of gene-disease associations divided by the total number of pieces of evidence.

**Categorization of DEGs by cell-type enrichment** Single-cell RNA sequencing data produced using mouse Hb were obtained from the GEO database (GSE13478). Clustering and cell type annotation were performed as described previously [3]. Differential expression analysis for each cell type was performed using the Wilcoxon rank-sum test as implemented in the “FindMarkers” function of the Seurat package (v.4.0.4). DEGs obtained from human microarray data were first converted to orthologous mouse genes using SynGo [4]. To examine cell type-specific enrichment, we compared the DEGs identified from microarray data with gene expression markers for each cell type (Log_2_FC > 0.25, adjusted p < 0.05). The average gene expression for each cell type was calculated and is listed in Table S3.

**Protein-protein interaction (PPI) analysis** A PPI network was created based on the Search Tool for the Retrieval of Interacting Genes (STRING) database [5] and visualized using Cytoscape 3.8.2. The confidence cut-off was 0.4. The subnetwork was analyzed using the cytoHubba plugin [6] with the maximum neighborhood component method.

**Gene ontology (GO) and transcription factor (TF) co-expression analyses** The list of DEGs was subjected to subsequent gene enrichment analysis using the web-based ENRICHR analysis tool (available at <https://maayanlab.cloud/Enrichr/>) [7, 8]. We particularly focused on the “Gene Ontology-Biological Process (GO-BP)” and “ARCHS4 transcription factor (TF) co-expression” options in ERICHR to predict significantly enriched biological functions and putative co-expressed TFs in the Hb tissues of suicides in comparison to unaffected controls.

**References for Materials and Methods**

1. Han S, Yang SH, Kim JY, Mo S, Yang E, Song KM, et al. Down-regulation of cholinergic signaling in the habenula induces anhedonia-like behavior. Sci Rep. 2017;7(1):900.

2. Gutiérrez-Sacristán A, Bravo À, Portero-Tresserra M, Valverde O, Armario A, Blanco-Gandía MC, et al. Text mining and expert curation to develop a database on psychiatric diseases and their genes. Database (Oxford). 2017;2017.

3. Hashikawa Y, Hashikawa K, Rossi MA, Basiri ML, Liu Y, Johnston NL, et al. Transcriptional and spatial resolution of cell types in the mammalian habenula. Neuron. 2020;106(5):743–758.

4. Koopmans F, van Nierop P, Andres-Alonso M, Byrnes A, Cijsouw T, Coba MP, et al. SynGO: an evidence-based, expert-curated knowledge base for the synapse. Neuron. 2019;103(2):217–234.

5. Szklarczyk D, Gable AL, Nastou KC, Lyon D, Kirsch R, Pyysalo S, et al. The STRING database in 2021: customizable protein-protein networks, and functional characterization of user-uploaded gene/measurement sets. Nucleic Acids Res. 2021;49:D605–D612.

6. Chin CH, Chen SH, Wu HH, Ho CW, Ko MT, Lin CY. cytoHubba: identifying hub objects and sub-networks from complex interactome. BMC Syst Biol. 2014;8 Suppl 4:S11.

7. Chen EY, Tan VM, Kou Y, Duan Q, Wang Z, Meirelles GV, et al. Ma'ayan A. Enrichr: interactive and collaborative HTML5 gene list enrichment analysis tool. BMC Bioinformatics. 2013;14:128.

8. Kuleshov MV, Jones MR, Rouillard AD, Fernandez NF, Duan Q, Wang Z, Koplev S, Jenkins SL, Jagodnik KM, Lachmann A, McDermott MG, Monteiro CD, Gundersen GW, Ma'ayan A. Enrichr: a comprehensive gene set enrichment analysis web server 2016 update. Nucleic Acids Res. 2016;44(W1):W90–7.
